# 

**Published:** 1883-09-20

**Authors:** 


RECEIPTED SUBSCRIBERS will be published in our next.

**NOTICE.**—*Many of our subscribers have forgotten us in the matter of remitting their dues. Friends, please attend to this matter at once. We are obliged to have money to run the Journal. Our printers are Cash men.* *W.*

### *EDITORIAL NOTICES.*

PROF. POWELL, our Senior Editor has returned from his summer jaunt at Blue Ridge Springs, Virginia, with health, flesh and spirits all improved. He now wants every *poor* Doctor in the land to take the RECORD, that they may learn of the benefits of Mineral water. The Doctor now modestly concedes what has been often said of him, that he is a "*big man.*" His present avoirdupoise is 220. W.

CHOLERA.—Reports of cholera indicate a subsidence of the disease at the points where it has prevailed, and it is now believed that we will escape the apprehended visitation of this dreaded scourge in the United States.

YELLOW FEVER is prevailing to some extent in the West Indies and in Mexico, but with the strict quarantine regulations now in force, and the fact that the season is well advanced, we may hope that our country will not be visited by the disease the present season.

PREMIUM OFFER FOR NEW SUBSCRIBERS.—See the premium offer of a beautiful and interesting monthly (THE CONTINENTAL) for new subscribers to THE RECORD. It may be found under our Special Notice head. The proposition will be open until further notice.

FREDRICK STEARNS & CO., DETROIT.—The advertisement of this great Drug House referred to editorially in our last, was unavoidably crowded out of that issue. Don't fail to examine their insert in the present number.

MEDICAL COLLEGES.—The whole number of medical schools in the United States, not including the Irregular schools, is put down at 49. Amongst these are three Female Medical Colleges—one at New York; one at Chicago, and one at Philadelphia. There are possibly others of which we have not heard. There is a medical college for colored people at Nashville, Tenn.

NO, SIR!—To the question, "Are the Editors of THE RECORD responsible for the sentiments, or do they vouch for the truth of statements made in the articles of their correspondents?" We reply that of course we are not so responsible. We give place oft times to opinions and statements which we do not endorse. Our columns are open to the free expression and interchange of opinion by the brethren of the profession, and we do not object to fair and honorable reply and criticism to anything found in our pages, but rather court it as a method of mutual instruction, and as a means of advancing medical science and developing the medical literature of the country.

### *PAMPHLETS RECEIVED.*

Some Remarks on Naso-Aural Catarrh and its Rational Treatment, by John N. MacKenzie, M.D., late House Physician in Bellevue Hospital, New York, and Chief of Clinic at the London Hospital for Diseases of the Throat and Chest, Surgeon to the Baltimore Eye, Ear and Throat Charity Hospital.

Neurotic Pyrexia, with special reference to Opium Addiction, by J. B. Mattison, M. D., Brooklyn, New York.

The Curability of Opium Addiction, by J. B. Mattison, M.D., Brooklyn, New York. Read before the King's County Medical Society, June 19th, 1883.

Clinical Notes on Opium Addiction, by J. B. Mattison, M.D., Brooklyn, New York.

A Personal Narrative of Opium Addiction, by J. B. Mattison, M.D., Brooklyn, New York.

Opium Addiction among medical men, by J. B. Mattison, M. D., Brooklyn, New York, read before the New Jersey Medical Society, Atlantic City, June 13th, 1893.

Observations on the management of Enteric Fever, according to a plan based upon the so-called Specific Treatment. Read before the College of Physicians of Philadelphia, January 3, 1883.

Club-Foot—Simple measures for its early relief, by DeForest Willard, M.D., Lecturer on Orthopædic Surgery in the University of Pennsylvania, Surgeon to the Presbyterian Hospital, etc.

Ambulance Service in Philadelphia. Read at the Academy of Music, April 30, 1883, by DeForest Willard, M.D., Surgeon to the Presbyterian Hospital, Lecturer on Orthopædic Surgery, University of Pennsylvania.

Adherent and Contracted Prepuce, commonly called Congenital Phimosis. Read before the Philadelphia County Medical Society, April 11, 1883, by DeForest Willard, M.D., Lecturer on Orthopædic Surgery in the University of Pennsylvania, and Surgeon to the Presbyterian Hospital.

